# Friction and Wear Behavior of Double-Walled Carbon Nanotube-Yttria-Stabilized ZrO_2_ Nanocomposites Prepared by Spark Plasma Sintering †

**DOI:** 10.3390/ma17153824

**Published:** 2024-08-02

**Authors:** Anne Kasperski, Dalya Alkattan, Viviane Turq, Claude Estournès, Christophe Laurent, Alicia Weibel

**Affiliations:** CIRIMAT, Université Toulouse 3 Paul Sabatier, CNRS-INP-UT3, Université de Toulouse, 118 Route de Narbonne, CEDEX 9, 31062 Toulouse, Franceclaude.estournes@univ-tlse3.fr (C.E.);

**Keywords:** spark plasma sintering, carbon nanotubes, zirconia, friction, wear

## Abstract

Double-walled carbon nanotube-yttria-stabilized ZrO_2_ nanocomposites are prepared by a mixing route followed by Spark Plasma Sintering. The double-walled carbon nanotubes (DWCNTs) have been previously subjected to a covalent functionalization. The nanocomposites present a high densification and show a homogenous dispersion of DWCNTs into a matrix about 100 nm in size. The DWCNTs are well distributed at the matrix grain boundaries but form larger bundles upon the increase in carbon content. The Vickers microhardness of the nanocomposites decreases regularly upon the increase in carbon content. Incorporation of carbon at contents higher than 2 wt.% results in significantly lower friction coefficients, both against alumina and steel balls, possibly because of the elastic deformation of the DWCNTs at the surface of the sample. Their presence also favors a reduction of the steel/ceramic contacts and reduces the wear of the steel ball at high loads. DWCNTs improve wear resistance and reduce friction without incurring any severe damage, contrary to multi-walled carbon nanotubes.

## 1. Introduction

The interest in all-solid self-lubricating materials, which eliminate the necessity of liquid lubricants, is well recognized. Carbon-containing nanocomposites, especially using carbon nanotubes (CNTs), are particularly noteworthy for their tribological applications [1,2]. Reports on the tribological properties of CNT–ceramic nanocomposites or coatings are abundant, in particular for the Al_2_O_3_ matrix [3,4,5,6,7,8,9,10,11,12,13,14,15]. The decrease in the friction coefficient was attributed to the spreading of carbon-based transferred films (tribofilms) over the contact area, allowing for easier shearing and providing lubrication during sliding. It has been proposed that the variations in friction behaviors could be linked to the thickness of the CNTs and the level of densification. The friction coefficient also shows a clear downward trend as the carbon content increases. This emphasizes that key points for achieving a higher microhardness, lower friction coefficients, and lower wear include high carbon contents, the homogeneity of the CNT dispersion, good interfacial bonding, and a high relative density. In spite of many potential applications, notably in the field of biomaterials, the friction behavior of CNT–zirconia nanocomposites is scarcely addressed [16,17,18] compared to the mechanical properties [19]. These studies are focused on yttria-stabilized zirconia (YSZ) in which either single-wall CNTs (SWCNTs) [16] or multi-walled CNTs (MWCNTs) [17,18] have been dispersed. An alternative way to the use of SWCNTs or MWCNTs for the preparation of self-lubricating CNT–ceramic nanocomposites is that of double-walled CNTs (DWCNTs), which are a unique class of CNTs [20,21], possibly more interesting for tribological applications. Indeed, it has been notably shown that the lubricating mechanisms depend on the number of walls and diameter of the CNTs: MWCNTs are cut and exfoliated, which leads to the formation of a lubricating film in the contact containing carbon debris, whereas DWCNTs have a better resistance to contact pressures and are elastically deformed [22]. The electrical and mechanical properties of spark plasma sintered DWCNT–YSZ nanocomposites have been published [23]. The aim of the present work is to investigate their tribological properties, which, to the best of our knowledge, have not yet been reported. It is shown that, by contrast to MWCNTs, the DWCNTs increase the wear resistance and reduce friction without incurring any severe damage.

## 2. Materials and Methods

### 2.1. Powder Preparation

A commercial nanometric (grain size slightly lower than 100 nm) 3 mol.% yttria-stabilized zirconia (3YSZ) powder (TZ-3Y, Tosoh, Tokyo, Japan) was used for the study. The proportions of tetragonal and monoclinic ZrO_2_ determined by X-ray diffraction (XRD) are 77 and 23 vol.%, respectively. DWCNTs were synthesized by a CCVD route [24]. The catalytic material designated as Mg_0.99_(Co_0.75_Mo_0.25_)_0.01_O was submitted to a catalytic chemical vapor deposition (CCVD) treatment (H_2_-CH_4_, 18 mol.% CH_4_, heating and cooling rates 5 °C·min^−1^, maximum temperature 1000 °C, no dwell), producing a CNT-Co/Mo_2_C-MgO nanocomposite powder. The powder was immersed in a 37% HCl aqueous solution in order to dissolve MgO along with the majority of cobalt and molybdenum species without damaging the CNTs [25]. The resulting suspension was filtered, washed with deionized water until neutrality, and kept wet (without any drying step) to facilitate further dispersion. The CNTs in the sample are mostly DWCNTs (80%), SWCNTs (15%), and CNTs with three walls (5%). The outer diameter is in the range of 1–3 nm, and the inner diameter is in the range of 0.5–2.5 nm [25]. The wet as-prepared DWCNTs were acid-functionalized using a mixture of nitric, sulphuric, and hydrochloric acidic solutions at room temperature [26]. The mixture was neutralized with ammonia and filtered while keeping the DWCNTs wet.

Five different DWCNT–3YSZ nanocomposite powders were prepared using the following route. The appropriate amount of acid-treated CNTs was dispersed in deionized water with a sonotrode (Vibra Cell 75042, Rosny-sous-Bois, VWR, France, 20 kHz, 500 W) for 15 min. The so-obtained CNT suspension was poured into a suspension of 3YSZ in water (pH = 12), which was prepared previously (15 min tip sonication and 1 h mechanical stirring). The mixture was then tip-sonicated for 30 min. The vessel containing the DWCNT–3YSZ suspension was immersed in liquid N_2_ until freezing and freeze-dried (Christ alpha 2–4 LD, Bioblock Scientific, Illkirch, France) at −84 °C for 48 h in a primary vacuum (12 Pa). The carbon content (C_n_) in the so-obtained DWCNT–3YSZ powders was measured by flash combustion (Perkin Elmer, Villebon-sur-Yvette, France, 2400 Series II) and is equal to 0.5, 1.2, 1.7, 4.5, and 6.3 wt.%.

### 2.2. Spark Plasma Sintering

The 3YSZ and DWCNT–3YSZ powders were densified by spark plasma sintering (SPS, Dr. Sinter 2080, SPS Syntex Inc., Kawasaki, Japan). A graphite die with a 20 mm inner diameter was loaded in the following order from bottom to top: a graphite punch, a sheet of graphitic paper, an alumina powder bed approximately 1.2 mm thick (in order to block the current and ensure uniform heating in specimens with varying electrical conductivities), another sheet of graphitic paper, the powder sample, and then the same materials in reverse order. The graphitic paper was also placed along the internal walls of the die to facilitate the easy removal of the pellets after sintering. SPS was conducted in an argon atmosphere using a conventional pulse pattern of 12–2 (12 current pulses followed by two periods of no current). The heating rate was 250 °C/min from room temperature to 600 °C, with a 3-min hold at 600 °C to stabilize the temperature reading. A heating rate of 100 °C/min was then applied from 600 °C to the target dwell temperature, either 1200 or 1350 °C, depending on the carbon content (Table 1), with a 10 min dwell period. A uniaxial load (equivalent to 100 MPa on the pellet) was gradually applied during the hold at 600 °C and maintained throughout the remaining heating and dwell period, then released in the final minute of the dwell. The cooling rate was set at 60 °C/min. The sintered specimens were formed into pellets 20 mm in diameter and approximately 2 mm thick, which were then polished to a 1 µm finish using diamond slurries. These sintered specimens will be referred to as 3YSZ, C0.5, C1, C2, C4.5, and C6 hereafter.

### 2.3. Characterization

Raman spectroscopy (Horiba Jobin-Yvon, Plaiseau, France, LabRAM HR800, 632.82 nm laser excitation) was used to characterize the raw DWCNTs, nanocomposite powders, the surface of sintered samples, and wear tracks, averaging at least three spectra for each specimen. X-ray diffraction (XRD, Bruker, Champs-sur-Marne, France, D4 Endeavor, Cu Kα radiation) was performed on sintered specimens. The density of the pellets was measured using Archimedes’ method after removing the graphitic surface contamination by polishing it with 600-grade SiC paper. Relative densities were calculated using 6.05 g/cm^3^ for tetragonal zirconia and 1.80 g/cm^3^ for DWCNTs, with a relative uncertainty estimated at 1%. The fracture surfaces of the pellets, coated with a 1 nm thick platinum layer, were examined using field emission gun scanning electron microscopy (FESEM, JEOL, Croissy, France, JSM 6700F). For each sample, the linear intercept method [27] was used to measure the size of a hundred 3YSZ grains. Indentation tests (3 N applied for 10 s in the air at room temperature) were performed on the polished surfaces of the specimens using a Vickers indenter (Shimadzu, Noisiel, France, HMV 2000). The calculated microhardness values (HV) are the average of ten measurements. Friction tests were conducted using a pin-on-disc reciprocating flat geometry (CSM Instruments, Peseux, Switzerland, Tribometer) in ambient air (30–36% relative humidity, 21–25 °C). Alumina and 100C6 steel balls 6 mm in diameter were used against the flat surfaces of 3YSZ and DWCNT–3YSZ samples. The sliding speed was set at 5 cm.s^−1^. Tests were performed with normal loads of 1, 5, and 10 N, depending on the ball used. Higher loads were not tested to avoid damaging the pellets and altering the contact geometry. The frictional force was recorded throughout the test using a load cell. Each friction test was repeated three times, yielding consistent results. Initial sample roughness was measured by white light interferometry (Zygo, Les Ulis, France, NewView 100). Wear tracks were analyzed using 3D optical profilometry (SENSOFAR, Terrassa, Spain, S neox) on the samples and optical microscopy (Keyence, Bois-Colombes, France, VHX-1000E) on the balls.

## 3. Results and Discussion

**Figure 1:**
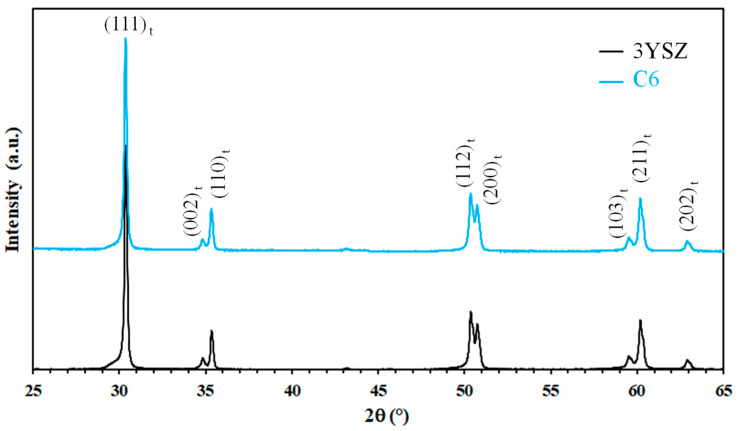
XRD patterns of the 3YSZ sample and the C6 nanocomposite.

**Figure 2:**
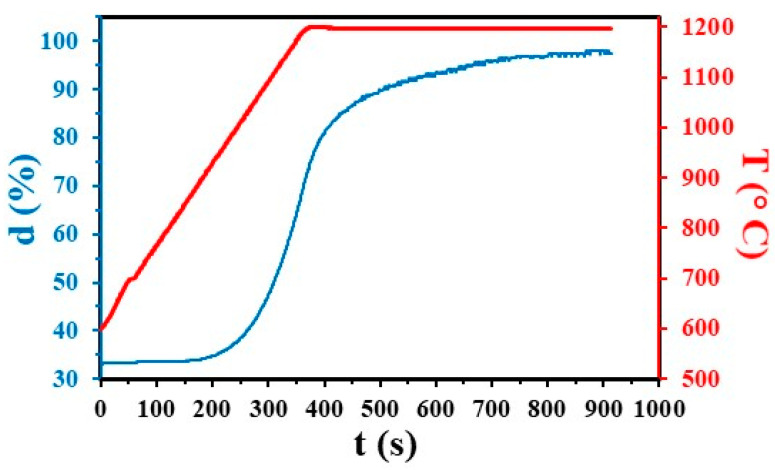
Evolution of the relative density and temperature during SPS of 3YSZ.

**Figure 3:**
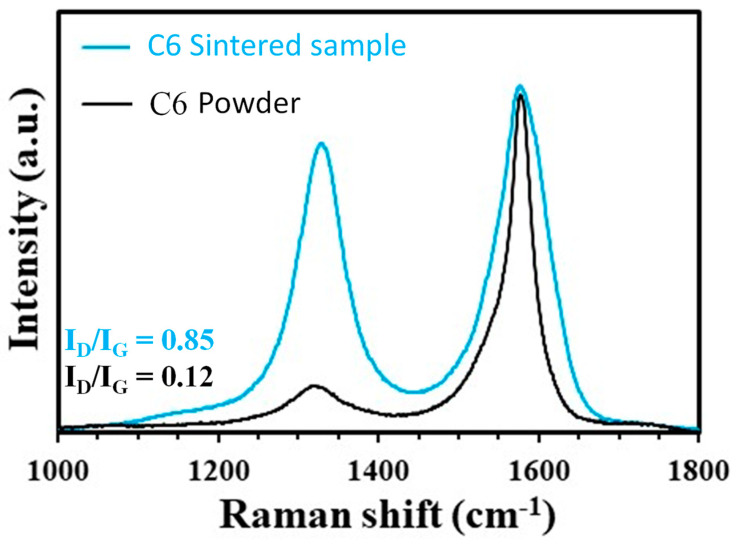
Raman spectra of C6 and corresponding nanocomposite powder.

Analysis of the XRD patterns (Figure 1) revealed only the presence of tetragonal zirconia in 3YSZ and C6. Similar patterns were obtained for all other sintered specimens, indicating that the presence of CNTs has no influence on the monoclinic to tetragonal phase transformation of 3YSZ during sintering. The densification occurs between 900 and 1200 °C for 3YSZ (Figure 2), in agreement with previous SPS studies starting from the same 3YSZ powder [28], and the relative density d reaches 98% at the end of the temperature dwell. The relative density (Table 1) is in the range of 98–100% for 3YSZ, C0.5, C1, C2, and C4.5 and is lower for the sample with a higher carbon content (C6), reaching only 96% despite the higher sintering temperature (1350 vs. 1200 °C). This agrees with earlier studies showing that CNTs above a certain proportion inhibit densification [29]. For all composite powders, the intensity ratio of the D band to the G band (I_D_/I_G_) in the high-frequency range of the Raman spectra (typical of the spectrum shown for the C6 powder in Figure 3) to that found for raw DWCNTs (0.11 ± 0.06 vs. 0.13) [23], indicating that the functionalization and mixing processes did not damage the DWCNTs. The higher I_D_/I_G_ ratio (Table 1) and the broadening of the G band attributable to the appearance of the D’ band for the sintered nanocomposites (typical spectrum shown for the C6 sample in Figure 3) compared to the powders could indicate that some DWCNTs were damaged during the SPS treatment. Ukai et al. [30] reported that the formation of zirconium carbide during hot-isostatic pressing of MWCNT/YSZ nanocomposites at 1450 °C during 2 h was responsible for MWCNT damage.

**Figure 4:**
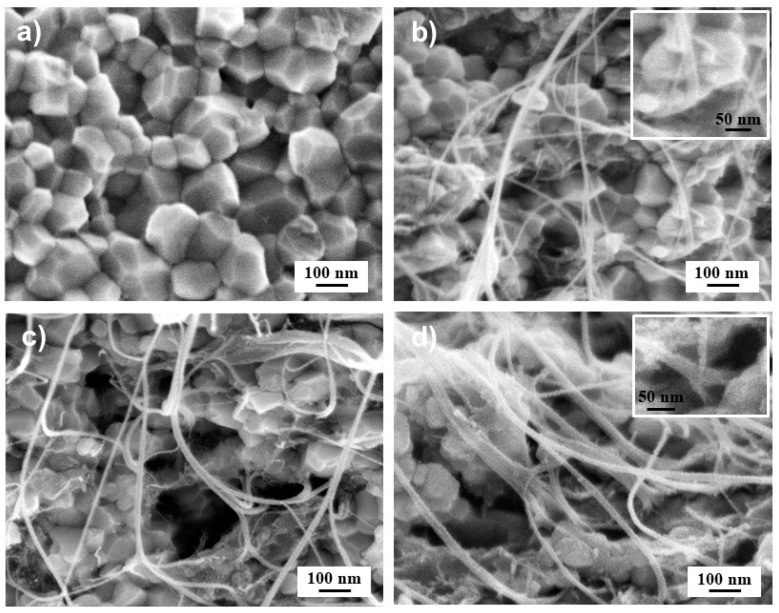
FESEM images of the fracture surface of the 3YSZ sample (**a**) and the C2 (**b**), C4.5 (**c**), and C6 (**d**) nanocomposites. The insets (**b**,**d**) are enlargements of the lower right parts of the corresponding images, highlighting stretched DWCNT bundles emerging from grain boundaries and anchored in the ceramic matrix.

However, no zirconium carbide was detected in the XRD patterns of the present sintered samples, possibly because SPS was conducted at lower temperatures (T ≤ 1350 °C) and for shorter durations (t = 10 min) in the present study. The potential for CNT damage during SPS may be more closely associated with the material’s mixed ionic–electronic conductivity at high temperatures [31]. Owing to their significant mobility, O^2−^ ions may react with the outer wall of the DWCNTs, especially since they are weakened by the covalent functionalization step [32], resulting in localized damage. For all samples, FESEM observations of the fracture surface (Figure 4) reveal little or no porosity, which is in agreement with the high relative densities (Table 1) and shows an intergranular fracture mode. The average grain size of the 3YSZ sample is equal to 100 ± 10 nm (Figure 4a), only slightly higher than in the starting powder. The same size (100 nm) is observed for the zirconia matrix for all samples, regardless of the carbon content. For C6, the high amount of DWCNTs hampered grain growth despite the higher sintering temperature (1350 vs. 1200 °C), thus accounting for the lower relative density, as noted above. For all specimens, the DWCNTs are well distributed at the matrix grain boundaries without forming agglomerates (Figure 4b–d). However, increasing amounts of DWCNTs lead to the formation of larger diameter bundles, up to about 100 nm (Figure 4b–d).

**Figure 5:**
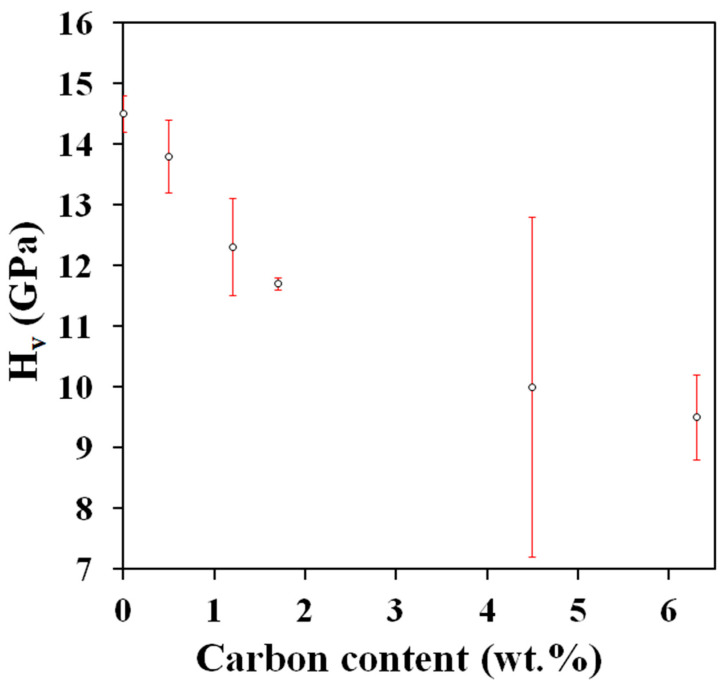
Vickers microhardness (H_v_) versus carbon content.

The Vickers microhardness of the nanocomposites (13.8–9.5 GPa, Table 1) is lower than that of the 3YSZ specimen (14.5 GPa, Table 1) and decreases regularly upon the increase in carbon content (Figure 5). The same behavior has been reported for fully densified SWCNT–3YSZ [33] and MWCNT–3YSZ [34] and is associated with a weak interfacial bonding between the CNTs and the zirconia matrix. The values obtained in this study are higher than those reported for SWCNT–3YSZ [16,33] and MWCNT–3YSZ [34,35], for which the composite powders were also prepared by a mixing route. The higher values could reflect a better dispersion of DWCNTs in the matrix and/or a slightly lower matrix grain size.

**Figure 6:**
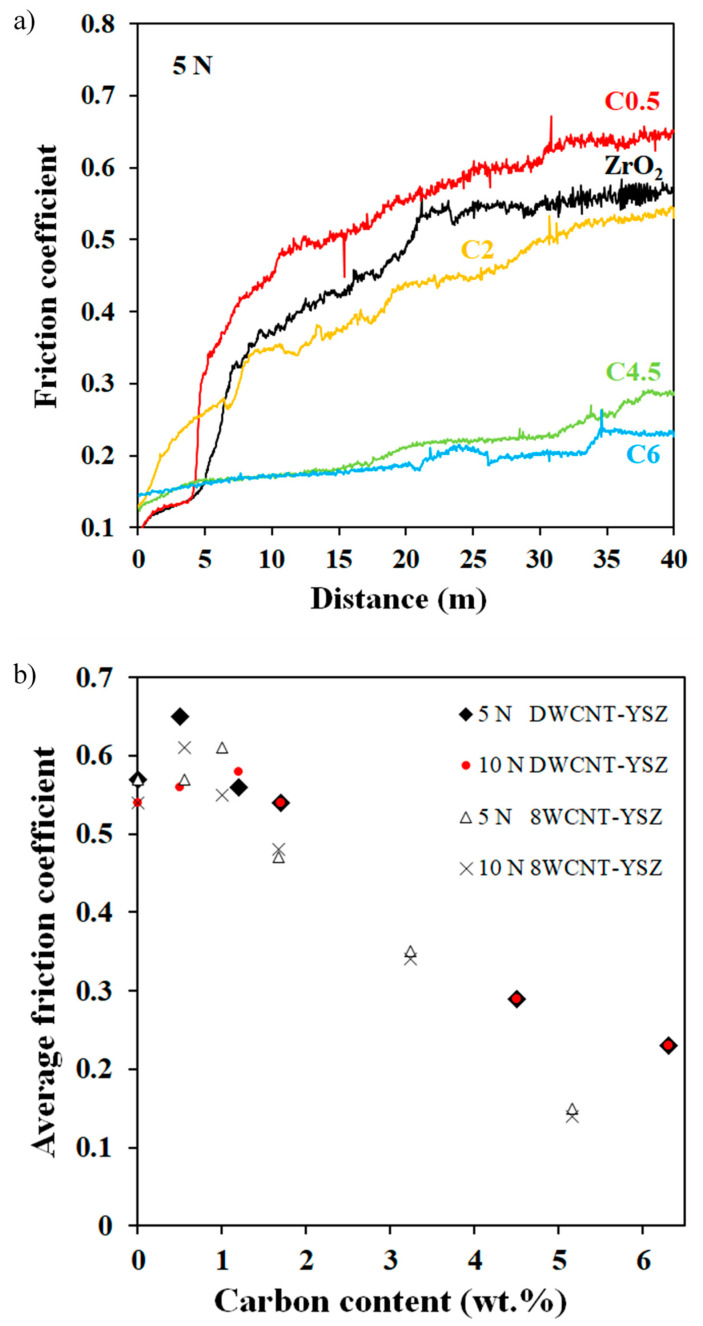
Friction coefficient against an alumina ball versus the distance for 3YSZ and DWCNT–3YSZ nanocomposites (**a**) and average friction coefficient against an alumina ball versus carbon content for DWCNT–3YSZ and 8WCNT–3YSZ [17] nanocomposites (**b**). The test load is indicated.

The arithmetic average roughness (Ra) calculated from white-light interferential rugosity images is equal to 0.01 µm for 3YSZ and is in the range of 0.05–0.07 µm for C2, C4.5, and C6 (Table 1). These higher values can be ascribed to the tearing of grains in the nanocomposites caused by the weakening of grain boundaries due to the presence of the DWCNTs. Typical curves showing the friction coefficient (µ) against the alumina ball versus distance for a 5 N load are shown in Figure 6a. For 3YSZ, C0.5, and C2, µ increases sharply during the running-in period and then stabilizes on the last 5 m. By contrast, for C4.5 and C6, µ increases smoothly and to a much lesser extent, with much less noisy curves. The observed noise reflects that the contact lacks stability and also a certain amount of wear, which is more pronounced for 3YSZ and the nanocomposites with low carbon contents. The alumina ball being harder (15 GPa) than the samples (9.5–14.5 GPa), wear can probably be attributed to that of the sample. The average friction coefficients calculated on the last 0.5 m versus carbon content are presented in Figure 6b. For the sake of comparison, earlier results on eight-wall carbon nanotube-yttria-stabilized ZrO_2_ nanocomposites (8WCNT–3YSZ) [17] are reported in Figure 6b. For loads of 5 and 10 N, µ decreases for carbon contents higher than 2 wt.% and reaches a value of 0.23, i.e., 2.4 times lower than for 3YSZ (µ ≈ 0.55). Thus, small amounts of DWCNTs probably weaken the 3YSZ grain boundaries but do not provide a lubricating effect, in agreement with results on SWCNT–3YSZ [15] and MWCNT–3YSZ nanocomposites [17,18,36].

**Figure 7:**
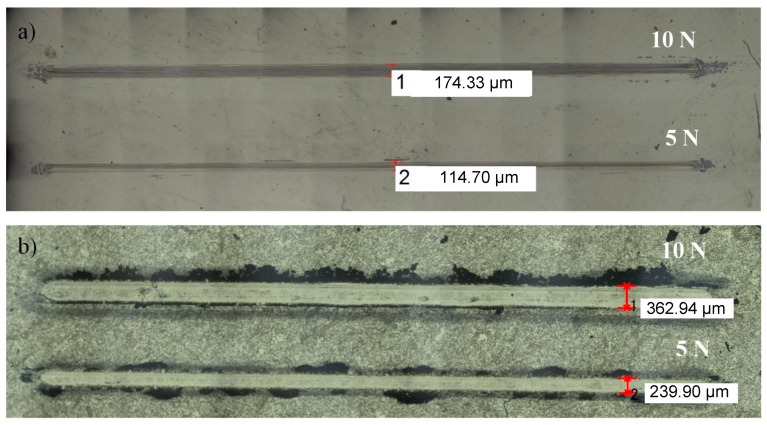
Optical micrographs of wear tracks after tests with an alumina ball at 10 and 5 N on 3YSZ sample (**a**) and C6 nanocomposite (**b**).

The reduction in the average friction coefficient starts at a lower carbon content for 8WCNT–3YSZ (2 wt.%) than for DWCNT–3YSZ (2 wt.% < C_n_ ≤ 4.5 wt.%) nanocomposites, probably because the pull-out of DWCNTs is more difficult than that of 8WCNTs, limiting their participation to the contact lubrication. Indeed, DWCNTs are longer (up to several tens of micrometers) than 8WCNTs (1.5 µm), and their significant sinuosity between the 3YSZ grains forms a network more firmly anchored in the matrix. The lowest value of µ (0.23) reached at both 5 and 10 N is lower than the one reported (0.35) by Hvizdoš et al. [37], who performed tests under similar experimental conditions (pin-on-disk test, alumina ball, 5 N, 25 m, room temperature, and dry conditions) on 3YSZ matrix nanocomposites containing 1.07 wt.% of carbon in the form of carbon nanofibers (CNF). Wear tracks on 3YSZ and C6 samples were observed by optical microscopy (Figure 7).

The track widths expand with the applied load, corresponding to the increase in contact radius calculated using Equation (1) and reported in Table 2:

a = (3FR/4E*)^1/3^ (1)

where F is the applied load (N), R is the ball’s radius (m), and E* is the equivalent Young modulus defined as follows:

1/E* = (1 − ν^2^_alumina_)/E_alumina_ + (1 − ν^2^_sample_)/E_sample_ (2)

where ν_alumina_ = 0.27, E_alumina_ = 390 GPa, ν_3YSZ_ = 0.31, and E_3YSZ_ = 212 GPa.

**Figure 8:**
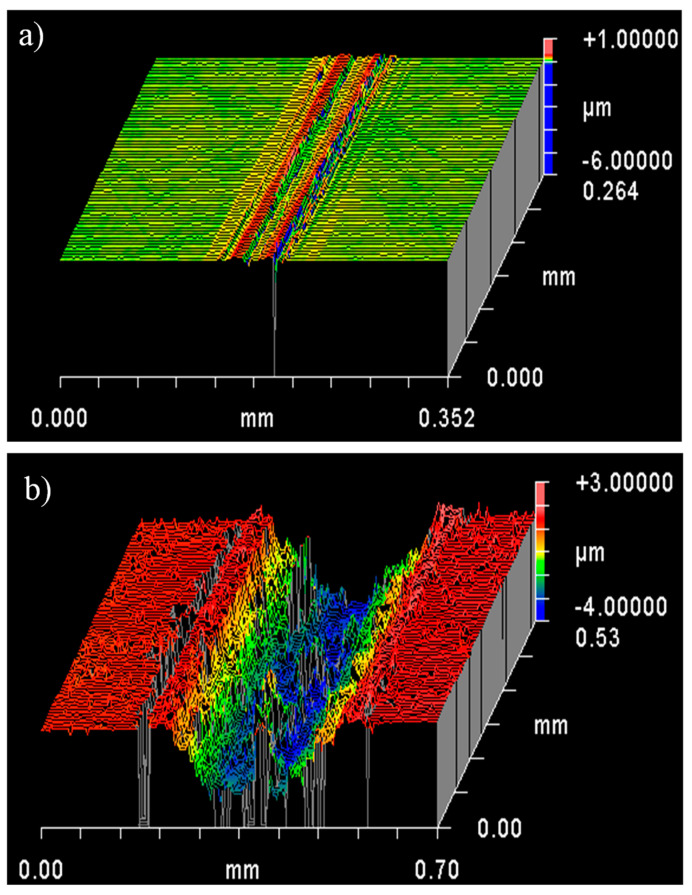
Wear tracks profiles after tests with an alumina ball at 10 N on 3YSZ sample (**a**) and C6 nanocomposite (**b**).

The track widths are approximately twice as large for C6 as for 3YSZ. Whether the test is conducted with a load of 5 or 10 N, the tracks on C6 are similar in appearance and can be identified by a significant amount of black debris, certainly containing carbon on the sides and less inside. The observation of the wear profiles (Figure 8) after tests at 10 N reveals a track with a depth estimated at 4 μm for C6, while it is superficial for 3YSZ, showing that the wear of the nanocomposite is much more significant than that of 3YSZ. For an alumina/C6 test compared to an alumina/3YSZ test, the reduction in the coefficient of friction is not correlated with a decrease in wear. Therefore, it seems that the threshold of carbon in the form of DWCNTs beyond which wear decreases has not been reached, contrary to what was observed for 8WCNT-based nanocomposites [17]. As with the difference in the coefficient of friction between 8WCNT–3YSZ and DWCNT–3YSZ nanocomposites, the difference in wear is likely attributable to a less easy DWCNT pull-out than 8WCNT pull-out.

**Figure 9:**
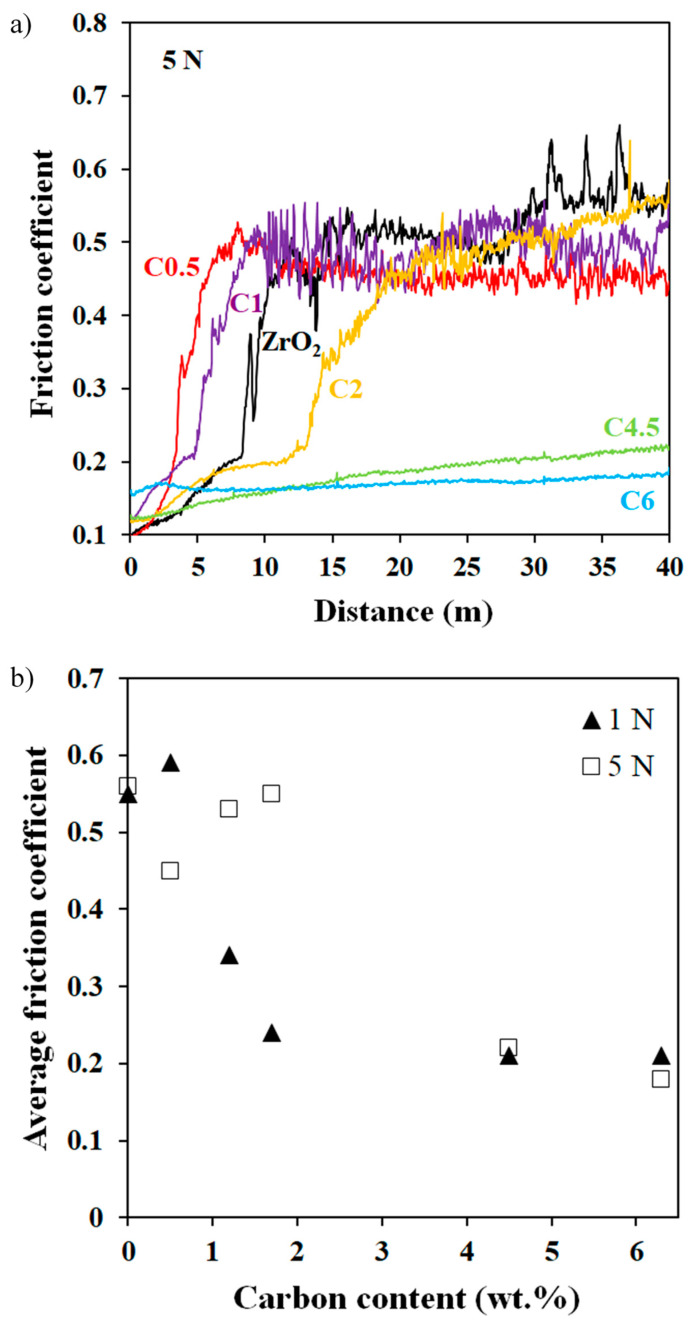
Friction coefficient against a steel ball versus the distance (**a**) and average friction coefficient against a steel ball versus carbon content (**b**) for 3YSZ and DWCNT–3YSZ nanocomposites. The test load is indicated.

**Figure 10:**
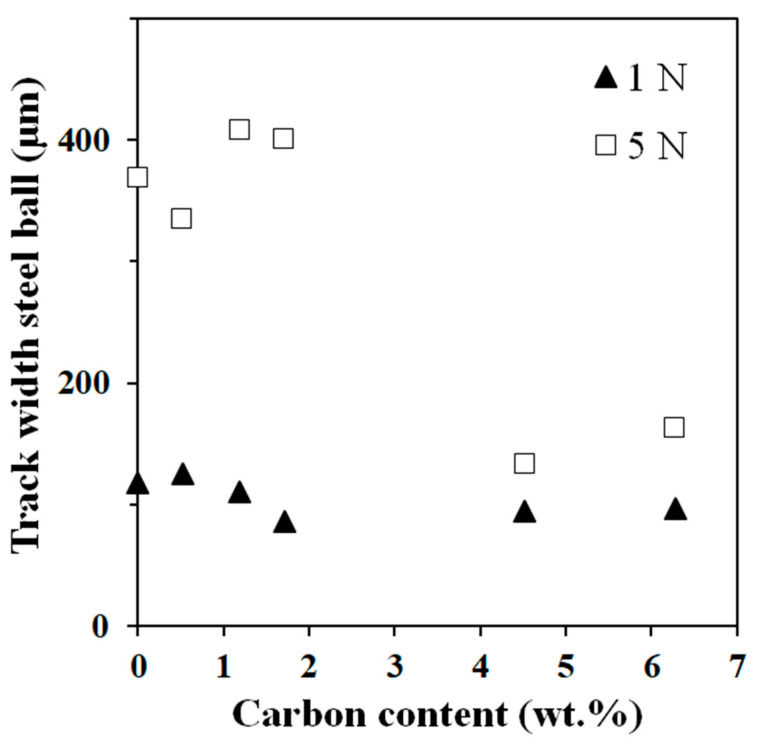
Track width on the steel ball versus carbon content for tests with DWCNT–3YSZ nanocomposites. The test load is indicated.

Typical curves showing the friction coefficient (µ) against the steel ball versus distance, for a 5 N load is shown in Figure 9a. For a load of 1 N against steel, µ starts decreasing for a carbon content above 1 wt.%., at least twice lower than at 5 N (Figure 9b). Beyond 2 wt.%, µ no longer depends on the load and carbon content. The lower value (about 0.2 for both C4.5 and C6) is almost three times lower than that for the steel/3YSZ pair. The steel ball being less hard (8.6 GPa) than the samples (9.5–14.5 GPa), wear can probably be attributed to that of the ball, and only the track width on the steel ball versus carbon content is presented (Figure 10). At 1 N, the wear track width on the steel ball remains constant at about 100 μm regardless of carbon content and is lower than those reported at 5 N, in agreement with the increase of the contact radius a (Table 2). At 5 N, wear track widths vary: those on steel balls in contact with samples with less than 2 wt.% of carbon are about 400 μm (similar to 3YSZ), while for C4.5 and C6 samples, they are less than 200 μm, showing significantly less wear. Moreover, this reduced wear is associated with lower and similar friction coefficients for C4.5 and C6 in comparison to other samples. The difference between the hardness of the ball (8 GPa) and the hardness of the nanocomposites (Table 1) decreases as the carbon content increases. This makes the contact between the two less aggressive, leading to a reduction in the wear of the ball with a 5 N load. Also, increasing carbon content in the form of DWCNTs appears to limit steel/ceramic contacts and improve the sliding of the ball.

The surface and wear tracks for C4.5 and C6 were analyzed using Raman spectroscopy after the tests with the alumina and steel balls. In each case, a signal corresponding to carbon appeared. The I_D_/I_G_ ratios calculated from the spectra (not presented) are similar, revealing no significant damage to the DWCNTs. According to Caillier et al. [38], DWCNT elastic deformation starts with a modification of the outer wall cross-section from circular to oval at above 80 MPa. The deformation of the inner wall into a peanut-like cross-section then occurs above 450 MPa. An increase of the pressure until 1 GPa did not reveal any irreversible deformation, while Aguiar et al. [39] reported a permanent deformation of the outer wall at about 21 GPa and of the inner wall at 25 GPa. The maximum Hertzian contact pressures sustained by the DWCNTs in the contact (P_max_—Table 2) were calculated using Equation (3):

P_max_ = 3F/2πa^2^ (3)

where F is the applied load (N) and a is the contact radius (m) calculated using Equation (1), ν_alumina_ = 0.27, E_alumina_ = 390 GPa, ν_steel_ = 0.33, E_steel_ = 200 GPa, ν_3YSZ_ = 0.31, and E_3YSZ_ = 212 GPa. 

The comparison of P_max_ with the values reported for DWCNTS [38,39] supports the Raman spectroscopy findings, indicating that the DWCNTs are elastically deformed under compression during friction. The DWCNTs are also submitted to shear strengths during the sliding of the ball. The maximum shear strength reached at the sample surface is calculated using Equation (4):

τ_max_ (MPa) = F_f_/A = µ_max_F/A (4)

where F_f_ is the tangential shearing force (N), µ_max_ is the maximum friction coefficient reached during the tests using four sets of conditions (alumina ball at 5 and 10 N, steel ball at 1 and 5 N), F is the applied load (N), and A is the contact surface area (m^2^) calculated using the contact radius a calculated using Equation (1) and reported in Table 2.

The calculated maximum shear strengths are in the range of 258–657 MPa (Table 2). Based on the average tensile strength of aligned DWCNT bundles, Li et al. [40] deduced that the average strength of an individual bundle is 6 GPa. An approximate value for the shear yield strength would be half of this or 3 GPa; therefore, it is significantly higher than the calculated τ_max_ values. Contrary to MWCNTs [17], DWCNTs are not severely damaged, cut, or destroyed to form a third lubricating body in contact, at least in the present experimental tribological conditions.

## 4. Conclusions

For the first time, a significant decrease in the average friction coefficient against both an alumina ball and a steel ball (by a factor of 2.4 to 3) is reported for DWCNT–3YSZ nanocomposites in comparison to 3YSZ. The decrease could result from the elastic deformation of the DWCNTs present at the surface of the sample. The presence of the DWCNTs also favors a reduction of the steel/ceramic contacts and reduces the wear of the steel ball at high loads. These tribological properties are achieved because of the specific microstructure of the nanocomposites, DWCNTs quality, DWCNTs homogeneous dispersion, low matrix grain size (100 nm), and sample high densification even for a relatively high carbon content (6 wt.%).

## Figures and Tables

**Table 1:** Carbon content in weight (C_n_), SPS dwell temperature (T_SPS_), relative density (d), Vickers microhardness (H_V_), I_D_/I_G_ (%) ratio between the D and G bands of the Raman spectra for the sintered nanocomposites and average arithmetic roughness (R_a_). Standard deviations are reported for H_V_. The I_D_/I_G_ ratios have been calculated from three to six spectra depending on the sample; the minimum and maximum values (min–max) are also reported.

Specimen	C_n_ (wt.%)	T_SPS_ (°C)	d (%)	I_D_/I_G_ (Min–Max)	H_V_ (GPa)	R_a_ (µm)
3YSZ	0	1200	98	-	14.5 ± 0.3	0.01
C0.5	0.5	1200	100	0.23 (0.09–0.72)	13.8 ± 0.6	-
C1	1.2	1200	100	0.74 (0.45–1.46)	12.3 ± 0.8	-
C2	1.7	1200	99	0.49 (0.37–0.61)	11.7 ± 0.1	0.05
C4.5	4.5	1200	98	1.07 (0.69–1.31)	10.0 ± 2.8	0.07
C6	6.3	1350	96	0.85 (0.33–1.20)	9.5 ± 0.7	0.06

**Table 2:** Ball and applied load (F) used for tribological tests, contact radius (a), maximum Hertzian contact pressure (P_max_), and maximum shear strength (τ_max_).

Ball	F (N)	a (µm)	P_max_ (MPa)	τ_max_ (MPa)
alumina	5	42	1346	586
	10	53	1696	657
steel	1	27	656	258
	5	46	1122	421

## Data Availability

The original contributions presented in the study are included in the article, further inquiries can be directed to the corresponding author.
